# Lived Experience and the Need for Co-Leadership in Mental and Substance Use Health Care

**DOI:** 10.1177/07067437251398100

**Published:** 2025-11-25

**Authors:** Tanisse Epp, Kim Hellemans, Kim Corace, Gord Garner, Benoit-Antoine Bacon

**Affiliations:** 1 Department of Neuroscience, 6339 Carleton University, Ottawa, ON, Canada; 2 CAPSA, Ottawa, ON, Canada; 3 113685 Canadian Centre on Substance Use and Addiction, Ottawa, ON, Canada; 4 Department of Psychiatry, 6363 University of Ottawa, Ottawa, ON, Canada; 5 Department of Psychology, 6339 Carleton University, Ottawa, ON, Canada; 6 Systemic Consulting Mental and Substance Use Health, Ottawa, ON, Canada; 7 Department of Psychology, 8166 The University of British Columbia, Vancouver, BC, Canada; 8 Institute of Mental Health, 8166 The University of British Columbia, Vancouver, BC, Canada; 9 Djavad Mowafaghian Centre for Brain Health, 8166 The University of British Columbia, Vancouver, BC, Canada

**Keywords:** co-leadership, lived experience, substance use health

It is by definition very difficult to fully understand and therefore to improve a system we have not experienced firsthand. As such, the knowledge of those who have navigated (or attempted to navigate) mental health and substance use health-care systems is essential to improving and rebuilding them.

There is definitely room for improvement. The almost certainly underestimated lifetime prevalence rate for substance use disorders in Canada is 20.7%^a^, with the prevalence of poor mental health at any time – again an underestimation – being 26.1%.^1^ Yet, only about one in 10 Canadians with a substance use disorder ever accesses formal services or supports^2^ and nearly eight in 10 individuals with mental health care needs report that those needs are unmet or partially met.^1^

The research is very clear: There are too many barriers to accessing care, including but not limited to shame, stigma, negative past experiences and structural challenges such as service availability, costs and transportation.^3–5^ These are the lived and living realities of many people in Canada, whose experiences could play a vital role in contributing to understanding these issues and reshaping every step of the journey through our inadequate mental and substance use health systems. However, these voices have been historically absent from decision-making bodies and leadership teams, contributing to persistent gaps in care, accessibility, and meaningful support.

Consider the difficult journey faced by the one out of 10 individuals who are seeking care for their mental or substance use health needs, a path not unfamiliar to several of the authors of this article. Before even reaching the threshold of care, individuals needing health care must first navigate the perspectives of family, friends and social media – all of which have their own ideas and opinions about ‘who’ and ‘what’ necessitates and deserves care.^6^ These social forces of influence shape perceptions and reinforce stereotypes, making it difficult for an individual to see themselves as someone in need or deserving of care.

As a result, the path to seeking care is rarely straightforward, in large part due to the pervasive stigma surrounding substance use. Stigma contributes to a fragmented system with numerous, often uncoordinated, points of entry. For example, an individual's first contact with the system may begin with a substance use medical emergency, involving paramedics or emergency department care providers, rather than through planned engagement with treatment or support services.^7^ Such emergency-driven entry is frequently the result of significant barriers at other access points, including uncertainty about where to begin or how to seek help.^8^ For example, it is estimated that for every 100 individuals who consume alcohol, one may be at high risk for early death due to their drinking, even though they never receive a formal diagnosis of alcohol use disorder.^9^ As a result, individuals who do not meet – or do not know if they meet – the diagnosable criteria, will only be able to interact with the care system once a crisis arises or after self-diagnosis where the focus will be on the disorder and not the individual's health.

Further perpetuating the barriers to care, negative attitudes and stigma toward people who use substances remain prevalent among health-care providers, indicating that the moral model of addiction persists well into the common era.^10,11^ Instead of feeling supported, individuals find themselves repeating their story to each new face, navigating a maze of disconnected services and sensing the subtle (or explicit) judgements that will shape their care. What is too often missing during this process is a sense of continuity, empathy, and genuine understanding. The absence of coordinated, person-centred support can leave individuals feeling invisible, misunderstood and unwelcomed, compounding the distress often led to seeking help in the first place, and unfortunately, resulting in individuals often receiving no help at all.

Internalized shame, guilt and negative attitudes towards one's substance use (i.e., self-stigma^12^) are often reflected in the common sentiment of feeling either ‘too sick’ or ‘not sick enough’ for care. These beliefs (often reinforced by systemic or structural stigma^13^) can lead individuals to view their own symptoms as either too severe or complex to be helped, or not serious enough to justify seeking care. Both of which can lead to delayed help-seeking or complete disengagement from care, driven by fear of rejection or substandard treatment from providers.^14,15^ Many, including some of the authors of this article, feel so unworthy that their conscious (or unconscious) beliefs suggest that they do not deserve care and that any efforts and resources would be wasted on them. For that one person out of 10 who gather the wherewithal to seek support for their mental and substance use health care, the result is too often a fragmented process shaped by public, structural and self-stigma, leading to *preventable* barriers to potential wellness.

So, if the goal is to make a more effective and efficient health-care system, the business case for integrating lived and living experience is clear: while it is certainly possible to study and analyse a health-care system from the outside, there are crucial insights that can only be gained through direct experience. Engaging those who have navigated (or attempted to navigate) mental and substance use health-care systems ensures that efforts to improve and rebuild these systems are informed by a comprehensive understanding of both the challenges and the opportunities.

While we often talk about ‘navigating’ the health-care system, this language can obscure the reality that, as we have highlighted, for many, there is no coherent system to navigate, only a series of broken pathways. Placing the onus on individuals to find their way assumes a logic and coordination that does not exist; instead, it is the responsibility of providers and policymakers to recognize and address these fragmented routes, and to work alongside those who have experienced them firsthand.

The integration of lived and living experience, using advisory and co-design models, improves clinical outcomes and program development, fosters mutual learning and empowerment, and leads to more person-centred care.^16–21^ Frameworks like Hart's Ladder of Participation^22^ and Youth Engagement Quality Standard^23^ support including those with lived experience at all organizational levels. While advisory and partnership models, like these, have advanced participation,^20,24,25^ they are often limited to specific projects or roles, restricting organizational impact and as such can often feel limited in impact to the people who are asked to serve. More direct, concrete and meaningful change can happen when people with lived experience are true co-leaders, involved at every level with shared authority and responsibility. Co-leadership is how we move from advisory roles to real influence, towards building mental and substance use health systems that are equitable, sustainable and impactful*.*

## What Is Co-Leadership?

Co-leadership is a collaborative approach where individuals with lived and living experience share equal authority and responsibility with institutional partners to shape health-care policies, practices, systems and research.^26^ This model is especially valuable in mental and substance use health, where systemic and social stigma have historically excluded people with lived and living experience from leadership and decision-making roles. In addition, with mental and substance use health challenges, the very conditions for which care is needed, makes it more challenging to seek and receive care.

Leadership teams have been shown to be most effective when they bring together diverse skills, backgrounds and experiences.^27^ In the context of mental and substance use health this means integrating the expertise of those working within the system with the insights of those accessing and receiving care, and those who have both expertise. Such diversity is not only a strength, but also essential for understanding and addressing the full complexity of the system, especially given the barriers created by stigma and fragmented systems.

A persistent, often invisible, barrier is the assumption that the people with lived and living experience can only contribute their personal stories. This narrow view can confine individuals to advisory roles, overlooking their broader expertise, education (both formal and experiential) and professional capabilities. Co-leadership moves beyond token representation, requiring organizations to recognize and utilize the full scope of skills, including analytical, organizational and leadership abilities that people with lived and living experience bring.

Much like an orchestra, where different musicians step forward at different times depending on the needs of the piece, effective co-leadership involves rotating leadership based on who is best equipped to guide at each stage, while making decisions together with clarity and respect. This approach demands clear agreements about roles, shared authority, and commitment to responsive, equitable engagement.^26^ Ultimately, co-leadership ensures that all participants are valued for their comprehensive expertise, not just their experience with the system, and creates a foundation for more responsive and effective care.

## Barriers to Realizing Co-Leadership

Despite its promise, co-leadership faces significant barriers. Persistent public, structural, and self-stigma barriers continue to marginalize people with lived and living experience, discouraging individuals to speak about their experiences with fear of negative consequences or ostracization.^27^ This stigma not only affects those seeking care but also exists within health organizations themselves. Many individuals working in health care have personally navigated the system for their own needs but choose to conceal their blended expertise to avoid judgment or professional repercussions, becoming a ‘hidden peer’.^28^ As a result, valuable insights from these individuals remain unheard, limiting the breadth of expertise within decision-making.

A fundamental barrier to realizing true co-leadership is the willingness of organizations and individuals to genuinely engage with and trust the insights and expertise of people with lived and living experience. This reluctance to share authority or decision-making power and to see this expertise as equal to clinical and academic experts reflects both cultural and structural stigma within existing systems and can impede the development of authentic, equitable co-leadership. Additionally, the concept of co-leadership remains unfamiliar in many organizations and within system decision-making practices. Most organizations and systems are not yet designed to facilitate engagement outside of traditional clinical roles or practices.^29^ Of the systems that incorporate those with lived and living experience, engagement is often symbolic or tokenistic, valuing only their story within the system rather than ensuring effective and meaningful participation with real decision-making authority.

## Co-Leadership in the Canadian Context

While it is still early days, many organizations have taken important first steps by integrating lived and living experience into advisory roles, policy discussions, and board participation. These organizations aim to ensure that people with lived and living experience have a formal place in shaping national conversations and priorities. While these structures may not yet reflect full co-leadership, they are helping build a foundation for more meaningful engagement in the future.

A standout example is AccessMHA in Eastern Ontario. AccessMHA was developed and implemented through a co-leadership model in which people with lived and living experience are not only consulted, but serve as co-leaders across all levels of governance, including system-level and oversight committees. This approach was foundational to the successful launch of this platform for coordinated access to mental and substance use health services in Eastern Ontario. The central principles of AccessMHA are that services must be both responsive to the needs of clients and families, and that people with lived and living experience are integral to every stage of design and implementation as leaders and partners.^29^

What sets AccessMHA apart is its collaborative governance structure, which facilitates genuine decision-making between people with lived and living experience and institutional partners, such as health-care organization leaders, clinicians and community representatives, beyond ‘advisory’ input. As a result, AccessMHA has been able to address long standing barriers, including fragmented care pathways, mismatched connections to care that does not meet individuals’ needs and lack of system responsiveness by co-leading solutions that reflect the needs and priorities of those who use the system. The success of AccessMHA demonstrates that co-leadership is not merely an aspirational ideal but a practical and effective strategy for system transformation.

It is important to acknowledge that many organizations are still navigating the transition from advisory or ‘voice’ roles to true co-leadership. Achieving this shift requires both structural and cultural change, including recognizing the broad range of skills and leadership capacity that people with lived and living experience bring.

## Call to Action

Building on these early successes, we call on all decision-making bodies and care providers in mental and substance use health care to move beyond representation and commit to genuine power-sharing with people with lived and living experience. The principle of ‘nothing about us without us’ must be more than a slogan, it should guide the transformation of our systems so that people with lived and living experience are not just present at the table but are true co-leads in decision-making at every level. This shift toward co-leadership is not just a matter of fairness; it is essential for overcoming the persistent challenges and limitations of our current ways of working. The status quo has left too many voices unheard and too many solutions unexplored. We need bold, systemic change and co-leadership is critical to achieving it.

We urge organizations to adopt structures that enable shared authority and accountability, and to transparently track and publish progress. In line with The Lancet's guidance, we call on researchers to prioritize diversity in authorship, including those with lived and living experience, and especially when working with historically marginalized groups, to clearly describe their approach to co-leadership and report the roles of all members involved throughout the research process.^30^ By doing so, we can empirically demonstrate the strength of this approach and inspire system-wide transformation.

## Conclusion

Integrating the blended expertise of people with lived and living experience into decision-making bodies and leadership teams is not a luxury or an after-thought, it is an absolute necessity for health systems to ensure equitable, effective and just practices and care. Co-leadership is the mechanism by which this integration becomes real, moving us from rhetoric to action. The question is not whether we can afford to do this, but whether we can afford not to. As we look to the future, let us commit to turning the barriers and hardships of the past into opportunities for partnership, hope, and lasting, effective change for the health of all people living in Canada.

## Notes

a. This prevalence estimate includes both substance ‘abuse’ and ‘dependence’. However, combining these categories misrepresents the distinct needs and challenges faced by individuals across the substance use health spectrum whose health may be impacted, including but not limited to those with substance use disorders. Co-leadership is necessary to address structural stigma and to accurately inform policy, research and service provision.
